# Abstracts from German Journals

**Published:** 1872-07

**Authors:** Ch. Raushenberg

**Affiliations:** Atlanta, Georgia


### ABSTRACTS FROM GERMAN JOURNALS.

BY CH. RAUSHENBERG, M.D., ATLANTA, GEORGIA.

*THE LOCAL MEDICAL TREATMENT OF HYPERTROPHIC TONSILS*

Was discussed at a meeting of the Medical Society of Berlin, December 13, 1871; and Dr. B. Fränkel, of Berlin, after alluding to the great inconvenience and danger frequently attached to this disease, and the impracticability of resorting to the surgical removal of the enlarged tonsils in some cases, advanced the following ideas in relation to their treatment without the knife. He mentions, for their local treatment—

1. *The caustics*, and amongst them, the chromic acid, as recommended by Dr. Lewin, of Berlin. Small pins of it are inserted into the tissue of the diseased gland. No pain and no reaction of any kind follows, but a very considerable shrinking of the tissue which the remedy penetrates. By frequent repetitions of this application, at regular intervals, and at different places of the tonsils, they can be reduced to one-half their size.

2. *The resolvent remedies, and amongst them, iodine, as the most prominent and effectual one.* The injection of the tincture of iodine into the central tissue of the gland has not disappointed Dr. B. Fränkel in a single case.

Before giving his own method, he refers to a publication of Dr. Franz Jakubowitz, of Nagy-Kàrvly (*Wiener Med. Presse*, Nos. 27, 28 and 29, 1871), in which four cases of chronic hypertrophy of the tonsils, and their successful treatment by injections of solution of iodide of potash (ten grains to one ounce), are related. Jakubowitz considers it of importance that the injections should reach the center of the tonsils, so that the injected fluid may come in contact with as large a portion of the tissue of the gland as possible, and that its escape, through one of the many follicular cavities which characterize the structure of the tonsils may be prevented. He also alludes to a paper of Rumboldt, who recommends injections of Lugol's solution—iod. 0.12 (about two grains), hydrojod. potass. 2.5 (about four grains), aquæ about one ounce—for submucous injections into the tonsils in the same affection; and considers two every week, and in all twelve to seventeen injections, necessary to accomplish the maximum result of this treatment, while Jakubowitz states that from ten to fifteen injections, made during a period of from four to six weeks, according to his experience, answer the purpose.

Dr. B. Fränkel himself uses a solution of iodine in glycerine, containing from one to two per cent. He depresses the tongue with a spatula in the left hand, and inserts the needle of the syringe deep into the hypertrophic tissue of the tonsil. No pain is connected with the operation, or follows it, unless the point of the needle has entered the surrounding muscular tissues. The hemorrhage is insignificant, and ceases without medical interference.

The difficulty connected with this method is caused by the peculiar structure of the tonsils. It happens but too easily that the point of the needle enters one of the many cavities penetrating the tissue, and then the injection, instead of remaining within, finds its way to the surface through the opening of the crypt it has entered.

Solution of iodide of potassium, on account of its want of color, almost excludes the possibility of observing this occurrence. Dr. Fränkel recommends the use of as fine a needle as possible, and presents, for inspection, a syringe which he has used in these cases, and which, in its plan of construction, favors those used by gynæcologists for uterine injections. He injects every eight days, and states that already, after the first injection, a considerable decrease in the size of the diseased tonsil takes place, but that from twenty to twenty-five injections are required to accomplish a diminution of the size of the tonsil to from one-third to one-fourth of its morbid volume.

In all cases where the tissue of a tonsil exhibits an unusual number of follicular cavities, and where, therefore, the retention of the injected fluid is difficult to accomplish, this peculiarity of structure causes Dr. Fränkel to resort to the application of iodine in a different manner. He uses these apertures for the point of insertion of as large a number of iodine pins (ten to twelve) into the tissue of the tonsils as their condition may permit. He has these needles similar in size and appearance to strong pins prepared out of equal parts of iodine and iodide of potash, with dextrin enough to obtain a mass of sufficient consistency to answer the purpose. He introduces about a dozen into the tonsils at each sitting, with the aid of a pair of pinchers. Some of these pins were produced to the Society for inspection, and their great solubility in water demonstrated.

A slight irritation of the tonsils sets in after their use; a yellow plug exudes from the cysts, and, with the same intermissions between their application as recommended in the use of the injections, a sufficient reduction of the tonsils is obtained in about the same length of time as by submucous injections of tincture of iodine.

In the discussion following these remarks, Dr. Lewin points to the necessity of distinguishing between *chronic congestion* and *chronic hypertrophy* (hyperplasia of Virchow) *of the tonsils.*

In the first, an enlargement of the vessels, combined with hyperæmia, exists; hence, even a very slight disturbance of the equilibrium between arterial influx and venus afflux of the blood causes a considerable exudation into the tonsils, which we all know under the form of acute tonsillar angina. The injuries caused by these painful and often highly inflammatory attacks, and their tendency to return frequently in consequence of the chronic congestion of the tonsils, are well known. Against this state of things, Dr. Lewin has used the chrystallized chromic acid, in substance, with very satisfactory results, as he can mention from sixty to eighty cases where, after its use, *he never heard of the return of the angina.* This acid, by giving off a portion of its oxygen, and being reduced to chromo-oxyd, causes a low degree of combustion, and hence ulceration of the tissue and an induration of the surrounding parts. The tissue becomes sclerotic, and seems to cause a compression of the enlarged capillaries, cutting off the possibility of inflammatory hyperæmia.

Dr. Lewin uses this occasion to mention the very beneficial effect of morphine injections into the central tissues of the tonsils, in acute painful conditions of these organs, in relieving pain and difficulty in deglutition.

In hyperplasia or hypertrophy of the tonsils, caused partly by a stiff exudate into the vascular membrane, between the epithelium and the fibrinous membrane, and partly by a hyperplasia of the lymphangoid tissue—a condition which generally impedes respiration, deglutition, and phonation—he has generally resorted to the surgical removal of the tonsils, but considers the treatment recommended by Dr. Fränkel, on account of its great technical applicability, very valuable.—*Berlin Klin. Wochenschrift, No.* 6, '72.

---

*THE IMPOSSIBILITY OF THE DIAGNOSIS OF SYPHYILIS BY MICROSCOPIC EXAMINATION OF THE BLOOD,*

In opposition to the opinions of Lostorfer, has been ably advocated by H. Köbner, of Breslau, in a lecture delivered, on the 12th of April last, before the Silesian Society of Home Culture, and contained at length in No. 18 of of the *Berlin Klinischer Wochenschrift*, 1872.

Köbner has followed Lostorfer's mode of preparing the blood for microscopic examination very carefully—has frequently seen the so-called syphilitic bodies of that observer, and considers them, in consequence of their chemical reaction, their great changeability in shape, and their entire want of permanent organic development, neither as fat corpuscles nor cryptogamic spores, as Wedl,* Lostorfer's first opponent, did. He has seen them in blood—treated with the utmost care, exactly as Lostorfer

*Wedl's Vortrag über die Lostorferschen Syphilis Korpuslin, in der Sitzung der k. k. Gesellschaft der Arzte, vom 9ter Febr. Allg. *Wiener Med. Zeitung,* 1872, No. 7.

requires it—from individuals laboring under variola, eczema, lupus and acne; he has seen no diminution of these bodies in syphilitic patients under energetic mercurial and iodine treatment. He has found them in the blood of healthy individuals, after from three to five days, and has, by ingenious changes in the mode of conservation of blood samples of all kind, established the following facts very nearly beyond a doubt:

1. That different modes of preservation, affecting the amount of air and moisture, and the degree of temperature acting on the blood destined for examination, have a greater influence on the microscopic changes it undergoes than its pathological origin. Thus he explains the correct diagnostic distinction made by Lostorfer between healthy and syphilitic blood, by a supposition that all the syphilitic blood samples received by him had been taken from the body much longer than those from healthy individuals.

2. That these so-called syphilitic bodies do not only make their appearance in the white, but also in the red corpuscles, and even in the plasma of the blood when it is exposed to a moderately higher temperature.

3. That they are the so-called vacuoli of modern botanists and zoologists, first described by Dujardin, and afterward minutely studied, by F. Cohn and Brown, in the cells of plants. They represent only very minute drops of water, of less tensity than the surrounding medium—hence, break the rays of light with less intensity; contain in solution salts, and perhaps other substances, and are surrounded by a very fine covering of albumen from the surrounding tissue.

4. That their appearance does not coincide with the conditions of putrefaction of the examined blood, but with those of a purely physical process—that of coagulation. All influences which he exerted, during the course of his investigations, to hasten or perfect coagulation, gave rise to an increase in the number of these bodies, and hastened their appearance; while those which retarded or prevented it (cold, addition of water, or water containing chloride of sodium in solution) had distinctly and plainly the contrary effect.

Corresponding with the process of putrefaction in the examined blood, he found the bacteria as microcoici and larcini—the latter very seldom—and both, almost with certainty, as intruders from without, as they were never observed unless the glue, fastening the covering of the preparations, had bursted.

